# Identification of Flower-Specific Promoters through Comparative Transcriptome Analysis in *Brassica napus*

**DOI:** 10.3390/ijms20235949

**Published:** 2019-11-26

**Authors:** Yan Li, Caihua Dong, Ming Hu, Zetao Bai, Chaobo Tong, Rong Zuo, Yueying Liu, Xiaohui Cheng, Mingxing Cheng, Junyan Huang, Shengyi Liu

**Affiliations:** Key Laboratory of Biology and Genetics Improvement of Oil Crops, Oil Crops Research Institute of Chinese Academy of Agricultural Sciences, Ministry of Agriculture and Rural Affairs, Wuhan 430062, Hubei, China; liyan@caas.cn (Y.L.); dongch@oilcrops.cn (C.D.); huming199217@126.com (M.H.); baizetao_2005@163.com (Z.B.); tongchaobo@gmail.com (C.T.); hu086zr@163.com (R.Z.); lyy680608@126.com (Y.L.); chengxiaohui@caas.cn (X.C.); mxchengcmx@163.com (M.C.); liusy@oilcrops.cn (S.L.)

**Keywords:** *Brassica napus*, Sclerotinia stem rot, petal, transcriptome, flower-specific promoter

## Abstract

*Brassica napus* (oilseed rape) is an economically important oil crop worldwide. Sclerotinia stem rot (SSR) caused by *Sclerotinia sclerotiorum* is a threat to oilseed rape production. Because the flower petals play pivotal roles in the SSR disease cycle, it is useful to express the resistance-related genes specifically in flowers to hinder further infection with *S. sclerotiorum*. To screen flower-specific promoters, we first analyzed the transcriptome data from 12 different tissues of the *B. napus* line ZS11. In total, 249 flower-specific candidate genes with high expression in petals were identified, and the expression patterns of 30 candidate genes were verified by quantitative real-time transcription-PCR (qRT-PCR) analysis. Furthermore, two novel flower-specific promoters (*FSP046* and *FSP061* promoter) were identified, and the tissue specificity and continuous expression in petals were determined in transgenic *Arabidopsis thaliana* with fusing the promoters to *β*-glucuronidase (*GUS*)-reporter gene. GUS staining, transcript expression pattern, and GUS activity analysis indicated that *FSP046* and *FSP061* promoter were strictly flower-specific promoters, and *FSP046* promoter had a stronger activity. The two promoters were further confirmed to be able to direct *GUS* expression in *B. napus* flowers using transient expression system. The transcriptome data and the flower-specific promoters screened in the present study will benefit fundamental research for improving the agronomic traits as well as disease and pest control in a tissue-specific manner.

## 1. Introduction

*Brassica napus* L. is an evolutionarily young allotetraploid species formed about 7500 years ago by natural hybridization between *Brassica oleracea* and *Brassica rapa*, followed by chromosome doubling [1,2]. *B. napus* is an economically important oil crop all over the world. It not only provides vegetable oil for people, but also provides high quality fodder for animals. In addition, owing to its favorable agronomic properties, such as cultivation under different seasons and rotation with cereals, *B. napus* is preferred by farmers worldwide.

However, Sclerotinia stem rot (SSR) is destructive to oilseed rape production. SSR is caused by *Sclerotinia sclerotiorum* (Lib.) de Bary, a cosmopolitan pathogen of many economically important crops. As a necrotrophic pathogen, it infects more than 600 plant species, including important oil crops such as oilseed rape, soybean, and sunflower [3,4,5,6]. SSR not only deteriorates the quality of the seed, but also significantly reduces the oil content [7].

Numerous studies have shown that flower petals play vital roles across the whole infection cycle of SSR, by supplying nutrition for ascospore germination and hyphae development [8,9,10]. As senescent petals fall onto leaves, petioles, and stems, secondary infection of SSR disease is initiated [8,9]. Therefore, the disease incidence is significantly positively correlated with petal infestation [9]. When the conditions for infection are favorable, *S. sclerotirum* causes severe yield losses in oilseed rape in the field [10].

Genetic engineering of crops, where resistant genes are introduced to improve crops resistance against disease, is a rapid breeding approach [11]. The cauliflower mosaic virus 35S promoter, actin, and maize ubiquitin promoters are the most commonly used constitutive promoters. However, these constitutive promoters trigger gene overexpression in all tissues, leading to some negative or undesirable pleiotropic effects, which interfere with normal gene expression and agronomic performance [12,13,14]. Tissue-specific promoters driving transgene expression within a given tissue are thus preferred. With control by these promoters, transgenes could result in spatio-temporal expression, which could eliminate unnecessary energy waste during normal plant growth and would make it easier to achieve precise and reliable results [15,16,17,18]. Consequently, specifically expressing resistant genes in oilseed rape flowers to control *S. sclerotirum* infection and extension will likely be effective.

To date, several flower-specific promoters have been reported and applied to research in the cut-flower industry. Most of these promoters are flavonoid synthesis-related gene promoters, and are used for altering corolla colors to create novel varieties in ornamental flowers, such as lily, petunia, and rose [19,20,21,22]. In Brassicacea, *Arabidopsis thaliana PISTILLATA* (*PI*) and *APETALA3* (*AP3*) are B-class organ identity genes in the ABC model, which are required for petal and stamen development [23,24,25,26]. At*PI* promoter driven GUS expression only occurs in the petals and stamens in *A. thaliana* [27]. However, the promoter of *B. napus* homolog gene *PISTILLATA-1* showed low flower specificity, under which GUS could also express in leaf and silique [28]. *BnQRT3* promoter drives GUS expressing in branch connective tissue between the pedicels and stem, besides in flowers [29]. The expression patterns of homologous genes between *A. thaliana* and *B. napus* are different, even though they all belong to Brassicacea. Consequently, it is necessary to identify new flower-specific promoters in *B. napus* per se. In this study, flower-specific promoters in *B. napus* were identified based on transcriptome data from 12 different tissues including root, stem, leaf, flower bud, and six dissected flower parts. Two novel strictly flower-specific promoters were cloned and experimentally confirmed by a GUS reporter gene expression system. The transcriptome data and the flower-specific promoters screened in the present study will benefit fundamental research for improving the agronomic traits as well as disease and pest control in a tissue-specific manner.

## 2. Results

### 2.1. Transcriptome Analysis and Identification of Tissue-Specific Genes

Twelve different tissues from *B. napus* line ZS11 were collected and used to construct libraries for RNA sequencing. After quality filtering, we obtained 18 million to 51 million clean reads for each tissue library (SRA accession: PRJNA474576). Through mapping to the *B. napus* genome database, 67.85 to 87.88% of the clean reads could be mapped. Over 12 million to 26 million unique reads were obtained, which occupied 51.02 to 75.54% of the mapped data (Table 1).

**Figure 1:**
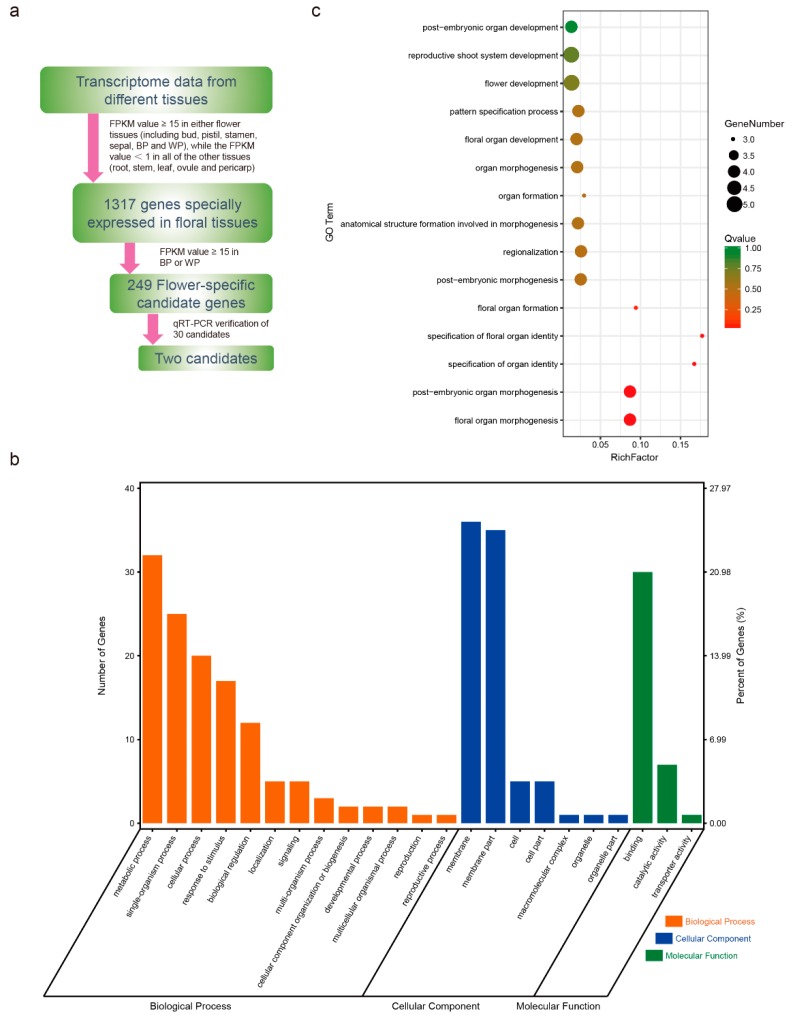
Flower-specific gene screening procedure and Gene Ontology (GO) classification and enrichment of flower-specific candidates: (**a**) Screening procedures for flower-specific candidate genes. (**b**) GO classification of flower-specific candidate genes. A total of 143 genes of 249 gene candidates have GO annotations. (**c**) GO enrichment related with organ formation, development or specification of flower-specific candidate genes. 31 GO terms were enriched, 15 of which were related with organ formation, development or specification.

The number of the tissue-specific genes were determined and summarized in Table S1. There are the most specific genes: 1074 genes in Flower bud, while the specific genes in the stem were the least: 38 genes. After filtering with high stringency based on the FPKM (fragments per kilobase of transcript per million mapped reads) value (Figure 1a), 1317 genes were detected as specifically expressed in floral tissues (including bud, pistil, stamen, sepal, lossomy petal (BP), and wilting petal (WP)). We further narrowed this to a list of 249 candidate genes that exhibited high expression in two kinds of petals (Figure 1a and Table S2). The 249 genes were regarded as flower-specific candidate genes. After Gene Ontology (GO) classification and enrichment using PlantTFDB 4.0 online tool, 143 genes had GO annotations (Figure 1b). Many genes were involved in the metabolic process of biological processes, and in the binding of molecular function. These results suggested that flower-specific candidate genes play different roles in flowers. Thirty-one GO terms were enriched, many of which were related with flower organ formation, development or specification. As shown in Figure 1c, five genes were related with flower development and reproductive shoot system development; four genes participated in pattern specification process, floral organ development and organ morphogenesis. These results indicated that the flower-specific candidates we screened were reliable.

### 2.2. qRT-PCR Analysis of Flower-Specific Candidate Genes

**Figure 2:**
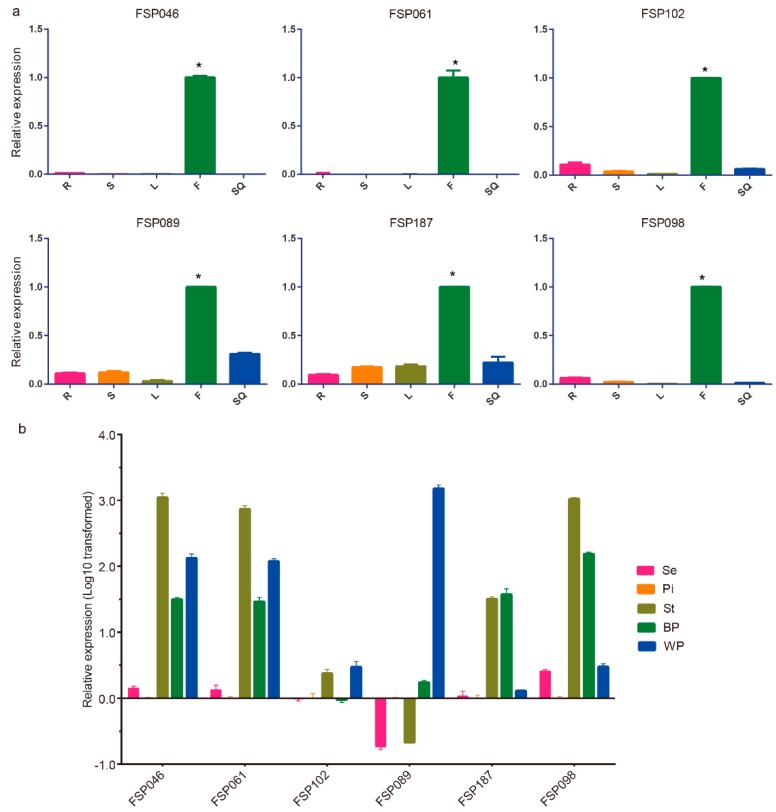
Expression profiles of six flower-specific candidate genes in different tissues by quantitative real-time transcription-PCR (qRT-PCR) analysis. (**a**) Expression patterns of six candidate genes in five different tissues. R (root), S (stem), L (leaf), F (flower bud), SQ (silique). The asterisk indicates that the expression value in the flower bud was at least three times higher than that in other tissues. (**b**) Expression profiles of six flower-specific candidate genes in dissected flower parts. The relative expression values were log10 transformed. Se (sepal), P (pistil), St (stamen), BP (blossomy petals), WP (wilting petals). Tissues from five plants were collected together, three technical repeats were performed and three biological repeats were conducted. The *B. napus β*-actin gene (*AF111812*) was used as a reference standard.

Thirty of 249 flower-specific candidates, most of which specifically expressed in BP or WP, were selected for quantitative real-time transcription-PCR (qRT-PCR) analysis. In the first round of detection, we defined flower-specific genes as those with expression values in the flower buds that were at least three times higher than that in other tissues (Figure S1). Six genes (*FSP046*—BnaA03g02790D, *FSP061*—BnaA03g18510D, *FSP102*—BnaC07g09080D, *FSP089*—BnaA07g11640D, *FSP187*—BnaUnng01380D, *FSP098*—BnaC01g10920D) exhibited specific and high expression in flower buds (Figure 2a). Then, the expression patterns of these six genes were detected in dissected flower parts (Figure 2b). To screen the continuous expression during the flowering stage, we detected the expression levels of the six genes in two kinds of petals (BP and WP) in detail. As illustrated in Figure 2b, five of the six candidates (excepting *FSP102*) were highly expressed in BP or WP, while only *FSP046* and *FSP061* were highly expressed in BP and WP at the same time. In addition, the expression levels of the four candidates (*FSP046*, *FSP061*, *FSP187*, and *FSP098*) in the stamen were relatively high.

### 2.3. FSP046 and FSP061 Promoters are Flower-Specific in Transgenic A. thaliana

**Figure 3:**
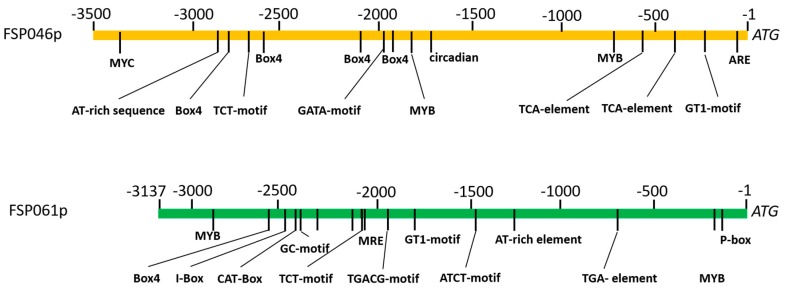
Conserved *cis*-elements in *FSP046* and *FSP061* promoters. Motif positions are indicated relative to the start codon.

To detect the flower-specificity, about 3.5 Kb of upstream sequences of two flower-specific candidate genes (*FSP046* and *FSP061*) from the ZS11 cultivar were cloned and sequenced. The *cis* elements in these two promoter regions were analyzed using the PlantCARE online tool; and the results are illustrated in Figure 3. One P-box, which contributes flower-specific gene expression, is found in the *FSP061* promoter sequence, but none are identified in the *FSP046* promoter sequence. There are several MYB, and MYC elements located in both promoter regions. Many *cis* elements related to light responsiveness, such as Box 4, I-box, TCT-motif, and GT1-motif, are found in both *FSP046* and *FSP061* promoters.

**Figure 4:**
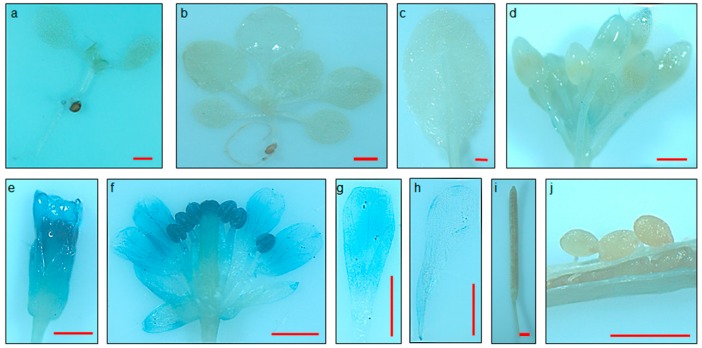
Histochemical staining of *FSP046::GUS* transgenic *A. thaliana* plants. There is no GUS staining in the two-leaf stage (**a**), six-leaf stage (**b**), rosette leaf (**c**), flower bud (**d**), silique (**i**), or seed (**j**). GUS activity driven by the *FSP046* promoter was observed in flowers one day before blooming (**e**), flowers at the blooming day (**f**), petals one day after blooming (**g**), and petals two days after blooming (**h**). Moreover, there is the highest GUS activity in the stamen/stigma (**f**), and less activity in the petals (**f**–**h**). Bar = 1 mm

To detect the tissue-specific expression, the whole upstream sequences of *FSP046* and *FSP061* gene were fused to the *GUS* reporter gene (*FSP046::GUS* and *FSP061::GUS*) and transformed into *A. thaliana*. Histochemical staining was detected during the two-leaf, six-leaf, and flowering stages of T2 generation plants by histochemical assay (Figure 4 and Figure S2). The GUS protein within *FSP046::GUS* transgenic plants was specifically expressed in flowers (Figure 4e–h). Moreover, there is the highest GUS activity in stamen (Figure 4f) and less activity in the petals (Figure 4f–h), which is in good accordance with *FSP046* gene expression pattern in *B. napus* flowers (Figure 2b). However, no GUS staining was detected in the roots (Figure 4a,b), leaves (Figure 4a–c), siliques (Figure 4i), or seeds (Figure 4j). The *FSP061::GUS* transgenic plants showed also flower-specific and similar GUS expression pattern (Figure S2).

**Figure 5:**
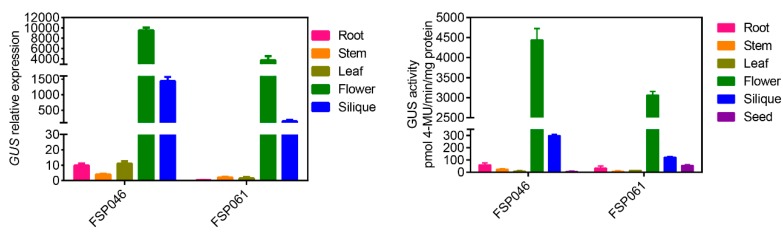
*GUS* gene expression in transgenic *A. thaliana* plants. (**a**) *GUS* gene expression patterns in five different tissues (root, stem, leaf, flower, silique) under *FSP046* and *FSP061* promoter direction. The *A. thaliana Actin* gene (*AT3G18780*) was used as reference gene. (**b**) GUS activity in six different tissues (root, stem, leaf, flower, silique, and seed) under *FSP046* and *FSP061* promoter direction. GUS activity was calculated as pmol 4-MU per min per mg protein. 4-MU, 4-methylumbelliferone.

To accurately define the tissue-specific expression pattern, we performed qRT-PCR analysis and GUS activity assays in transgenic *A. thaliana* (Figure 5). The *GUS* gene expression was exceedingly high in flower comparing with that in root, stem, and leaf. Moreover, the expression levels of the *GUS* gene in the flower were about seven and 25 fold higher than that in silique under *FSP046* and *FSP061* promoter, respectively (Figure 5a). The GUS activity results also showed the same expression pattern among different tissues (Figure 5b). Furthermore, we also detected GUS activity in transgenic *A. thaliana* seeds. Of which the activity was obviously lower than that in silique. In summary, *FSP046* and *FSP061* promoter could drive *GUS* specifically expressed in flowers, and the activity of *FSP046* promoter was higher than that of *FSP061* promoter.

### 2.4. FSP046 and FSP061 promoter can Drive GUS Expressing in Flowers of B. napus

**Figure 6:**
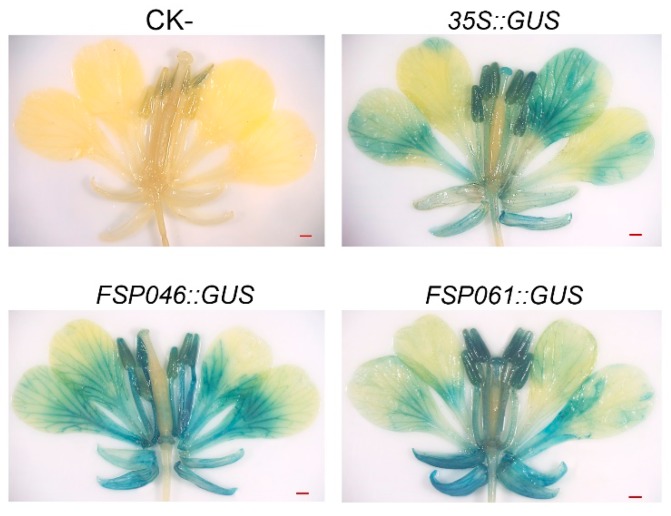
Histochemical staining of *B. napus* flowers under transient expression. GUS staining can be observed in flowers of the positive control *35S::GUS*, *FSP046::GUS*, and *FSP061::GUS*, respectively (especially in their sepals, petals, and stamens). While no GUS stained could be detected in negative control (CK-, the empty *Agrobacterium*). Bar = 1 mm.

Based on the flower-specific expression in transgenic *A. thaliana*, we further investigated whether the *GUS* gene could be expressed under *FSP046* and *FSP061* promoter driving in *B. napus* flowers. Using the transient expression system, we detected that both of the flowers can be stained by GUS reaction buffer. As shown in Figure 6, comparing with inoculating empty *Agrobacterium*, the sepals, petals, stamen, and stigmas of *FSP046::GUS* and *FSP061::GUS* could be clearly stained, which was similarly found for the *35S::GUS* positive control transiently transformed flowers. These results confirmed that both promoters can drive GUS expressing in flowers of *B. napus.*

## 3. Discussion

In genetic engineering, using tissue-specific or temporal-specific promoters to drive gene expression can avoid unwanted influences on plant phenotypes caused by constitutive promoters [17,18,30]. To find tissue-specific promoters, researchers usually identify the genes expressed in a tissue-specific manner. Previously, gene expression was typically analyzed by subtractive hybridization [31] and gene expression microarray [32], which were time consuming or had high background noise. In contrast, RNA-seq is a low-cost and high-throughput sequencing technology for genome-scale gene expression analysis [33]. Analyzing transcriptome data from different tissues to obtain tissue-specific genes is a high throughput, practical, and rapid method, which has been applied in peanut and poplar [34,35]. In the present study, we sequenced the transcriptomes of 12 different tissues from *B. napus*. After analyzing the data, 249 flower-specific genes highly expressed in petals were identified, and two novel flower-specific promoters were selected for cloning and confirmed to be flower specific through driving *GUS* reporter gene expressing in transgenic *A. thaliana* plants.

In present study, both *FSP046* and *FSP061* promoters were proved to be flower-specific. It was reported that the P-box contributes to flower-specific gene expression of *gPAL2* of *Phaseolus vulgaris* [36], *chsA* of *Petunia hybrid* [37] and *chs* of *Phalaenopsis hybrid* [38]. However, only the *FSP061* promoter sequence contained the P-box (sequence AACCAAAC) according to the P-box consensus sequence [37]. Therefore, we presumed that there may be other unknown motifs contributing to flower-specific expression in *B. napus*. In future studies, GUS activity of different deletions of the two promoters will be implemented to find novel flower-specific motifs, and this method will also be used to identify the core regions of *FSP046* and *FSP061* promoters.

From Figure 4 and Figure S2, the stamen was deeply stained by GUS reaction buffer, and exhibit higher GUS activity than petal. *AtPI* and *AtAP3* promoters also drive GUS expressing in petal and stamen [26,27]. Oilseed rape XY355 was reported as a petal-specific promoter [39], while *XY355::OvPAP2* transgenic *B. napus* plants exhibited red petals and stamen [40]. It was worth noticing that genes that were expressed in petals were usually accompanied by expression in stamen. Up to now, there is only one promoter (*InMYB1* promoter) driving GUS protein expression in petals distinctly and uniquely in *A. thaliana* [41]. Young and Werner reported that stamens were probably an important infection route of *S. sclerotiorum* for apetalous winter oilseed rape [42]. Consequently, *FSP046* and *FSP061* promoters are suitable to apply in SSR disease control in a flower-specific manner.

In transgenic *A. thaliana*, both of the *GUS* gene expression level and protein activity in *FSP046::GUS* transgenic plants were higher than that of *FSP061::GUS* in flowers (Figure 5). These results were consistent with the qRT-PCR results in *B. napus* (Figure 2b), which indicated that the *FSP046* promoters have stronger activity to drive downstream gene expression. In addition, from Figure 5a, the *GUS* gene could also be detected in silique with a relative higher expression level. To further detect whether *GUS* was expressed in transgenic seeds, we directly assayed the *GUS* activity, and found that the activity were decreased by about 98% and 54% in *FSP046::GUS* and *FSP061::GUS* transgenic seeds, respectively (Figure 5b). These results indicated that *FSP046* and *FSP061* promoters have a strict flower specificity, which were more safety to environment for application as the flower is senescent tissue.

In the present study, we use the syringe-press transient expression system to over-express *FSP046::GUS* and *FSP061::GUS* in *B. napus* flowers. Using the transient expression system, all flowers (especially sepals and petals) could be stained by *GUS* reaction buffer (Figure 6), which indicated that both promoters can drive *GUS* expression in *B. napus* flowers. Compared with hypocotyl transformation, the transient expression system in flowers was fast and efficient, which could also be applied in subcellular localization, BiFC assay, and gene function analysis of *B. napus* genes.

The multiple functions of oilseed rape (including seed oil, vegetable bolt, flower sightseeing, and fodder for animals) are heavily researched. Many regions in China have constructed large-scale oilseed flower sightseeing tourism, which has become an important part of the local economy [43]. To add aesthetic value, genetic engineering under the direction of flower specific promoters is a timesaving way to produce rich flower colors of oilseed rape. Furthermore, the flower-specific promoters isolated in this study could also be used to modify colors for ornamental flowers such as lily and chrysanthemum. SSR is the major threat for oilseed rape production, and it was reported that pollen beetle (*Meligethes aeneus*) was a major pest at the inflorescence stage [44]. In recent years, cross-kingdom RNAi has been discovered between host plant and pathogens, pests [45,46,47]. Host-induced gene silencing (HIGS) by transgenic expression of pathogen/pest gene-targeting double-stranded (ds)RNA is a promising alternative to control disease/pest in plant protection [48]. Our flower-specific promoter has the potential to be combined with the HIGS technique to control SSR and pests in a tissue-specific manner.

## 4. Materials and Methods

### 4.1. Plant Material Collection

Seeds of *B. napus* line ZS11 were obtained from Key Laboratory of Biology and Genetic Improvement of Oil Crops at OCRI and sown in October in the field located in Wuhan (average annual temperature 13–22 °C and 70–80% humidity), Hubei province, China. Hubei province is the major production area for winter rapeseed planting in the middle and lower Yangtze River. The trial management followed standard breeding field protocols. Tissue samples were collected at the full-bloom stage in the following spring. Root, stem, leaf, flower bud, little siliques (about 4 cm long), and dissected flower parts including blossomy petal (petal at the blooming day, BP), wilting petal (one day after blooming, WP), pistil, stamen, sepal, ovule, and pericarp were collected from 15 plants in the same developmental stage in the same test plot.

### 4.2. RNA Preparation and RNA-seq Analysis

Total RNA was extracted from different tissues using TRIzol reagent (Invitrogen, Carlsbad, CA, USA). Poly-A-containing mRNA was isolated from the total RNA using Oligotex mRNA Mini Kit (QIAGEN, San Francisco/Bay area, CA, USA) according to the manufacturers’ instructions. RNA libraries with insert sizes of 250 bp were constructed for each tissue sample and sequenced on an Illumina HiSeq 2000 platform (San Diego, CA, USA) at BGI Co. Ltd. The libraries were sequenced for paired-end reads of 150 bp.

### 4.3. Functional Annotation

The raw reads were filtered to remove adapter sequences, low-quality reads, and reads containing poly-N using the tools from the NGS QC Toolkit (v2.3) [49]. The derived clean reads were mapped to the reference genome of *B. napus* line Darmor-*bzh* (*Brassica napus* Annotation v5, http://www.genoscope.cns.fr/brassicanapus/) [1] using TopHat software (v2.0.9) [50]. The mapped reads were quantified by fragments per kilobase of transcript per million mapped reads (FPKM) for each gene using Cufflinks (v2.1.0) software [50].

### 4.4. Screening of Tissue-Specific Candidate Genes

Tissue-specific genes were screened and calculated using the threshold of FPKM value >2. We defined flower-specific genes as genes with threshold FPKM values ≥15 in either flower tissues (including bud, pistil, stamen, sepal, BP, and WP), while the FPKM values were <1 in all of the other tissues (root, stem, leaf, ovule, and pericarp). Furthermore, according to the trait of *S. sclerotiorum* tending to infect the plant petals primarily in the SSR disease cycle, the genes with FPKM values ≥15 in BP or WP were finally selected as flower-specific candidates. GO functional annotation and classification of the flower-specific candidate genes were conducted in the PlantTFDB 4.0 online tool (http://planttfdb.cbi.pku.edu.cn/) with the threshold of *p*-value ≤ 0.01 [51,52]. The GO enrichment result was displayed using the ImageGP online tool (http://www.ehbio.com/ImageGP/).

### 4.5. Quantitative Real-Time PCR Analysis

To verify the tissue specificity of flower-specific candidate genes, qRT-PCR primers of flower-specific candidate genes were designed (Table S3). Primer specificity was examined by PCR and agarose gel analysis. Total RNA was extracted using TRIzol reagent (Invitrogen, Carlsbad, CA, USA). About 1 μg of total RNA was reverse transcribed using the PrimeScript™ RT reagent Kit with gDNA Eraser (TaKaRa Co., LTD, Beijing, China). To screen the genes continuously expressed during the flowering stage (from flower bud to WP), the expression patterns of flower-specific candidate genes were first detected in five tissues including root, stem, leaf, flower bud, and silique by qRT-PCR analysis. The genes with specific expression in flower bud were used for subsequent qRT-PCR in dissected flower parts including sepal, pistil, stamen, BP, and WP. Three biological repeats were conducted. The *B. napus β*-actin gene (*AF111812*) was used as a reference standard. The relative expression was calculated using the 2^−∆∆*C*t^ method [53].

### 4.6. Cloning and Characterization of Flower-Specific Promoters

About 3.5 Kb sequences upstream of the start codon ATG were selected, and the primers (Table S3) were designed according to *B. napus* line ZS11 genome [54]. To analyze the significant cis elements of the flower-specific candidate genes, accurate promoter sequences were analyzed using the PlantCARE online tool (http://bioinformatics.psb.ugent.be/webtools/plantcare/html/) [55].

### 4.7. Binary Vector Construction and Plant Transformation

To construct the promoter::GUS reporter system, flower-specific gene promoters were cloned into pBI121 at HindIII and BamHI sites to replace the *CaMV 35S* promoter using the ClonExpress II One Step Cloning Kit (Vazyme Biotech Co., LTD, Nanjing, China). The primer pairs are listed in Table S3. Recombinant plasmids were introduced into *Agraobecterium tumefaciens* GV3101 competent cell (AngYu Biotech Co., LTD, Shanghai, China). *A. thaliana* transformation was performed using the floral dipping method [56]. For transient expression in flowers of *B. napus*, a syringe-press method was used for *Agro*-infiltration [57]. Briefly, the *Agrobacterium* cells were harvested and adjusted to OD_600_ 2.0. The resuspension solutions were put into a 50 mL syringe, and then, 10–15 flowers from *B. napus* line Westar were added into the solution. Positive pressure was used for 30 s, and the inoculated flowers were placed into wet box for preserving moisture. Forty-eight hours later, the flower tissues were collected for GUS staining. Three independent biological repeats were performed.

### 4.8. Histochemical GUS Assays and Evaluation of GUS Activity

Histochemical detection of GUS staining was performed as described by Jefferson et al. [44]. Transgenic positive *A. thaliana* seedlings, organs, and tissues from different T2 generation lines and flowers of *B. napus* were infiltrated in GUS reaction solution (Coolaber Science & Technology Co., LTD, Beijing, China) for 12 h at 37 °C in the dark. Several washes of 70% ethanol were performed to stop the GUS reaction and remove chlorophyll at room temperature. Then the stained tissues were observed under an anatomical lens (Olympus SZX16, Ac Adapter, Tokyo, Japan).

GUS activity from different transgenic positive *A. thaliana* tissues was measured according to Jefferson et al. [58] by measuring 4-methylumbelliferone (4-MU) fluorometric quantity. Different tissues were grounded to a finely pulverized powder in liquid nitrogen, and extracted in GUS extraction buffer. The protein concentration was measured with the Bradford method [59]. Fluorescence values were recorded with Glomax^®^ Explorer Multimode Microplate Reader (Promega, Madison, WI, USA). The GUS enzyme activity was calculated as picomoles of 4-MU produced per milligrams of protein per minute. The *GUS* gene expression in different tissues was examined by qRT-PCR analysis. The *A. thaliana actin* gene (*AT3G18780*) was used as a reference gene. The used primers were listed in Table S3.

## 5. Conclusions

In the present study, we sequenced the transcriptomes of 12 different tissues from *B. napus*, which provide a foundation for the expression patterns of gene family studies. Moreover, based on the RNA sequencing data, other tissue-specific promoters (such as in the root and stem) could also be screened using similar procedures, which could be used for disease and pest management in a tissue-specific manner, for example with clubroot disease and aphids. Therefore, the transcriptome data present in this study will provide practical guidance for other studies. In conclusion, the transcriptome data and the flower-specific promoters screened in the present study will benefit fundamental research and disease and pest control in a tissue-specific manner.

## Figures and Tables

**Table 1:** Transcriptome statistics of twelve different tissues from *Brassica napus.*

	Root	Leaf	Bud	Silique	Stamen	Pistil	Blossomy Petal *	Wilting Petal **	Stem	Sepal	Ovule	Pericarp
Clean data	47,855,967	51,669,765	50,250,950	47,583,789	20,822,443	23,301,853	24,872,044	23,779,169	18,987,585	25,334,038	23,375,360	25,707,408
All data mapping to genome	34,126,090	35,693,473	34,969,636	32,285,601	18,163,417	20,477,668	20,852,722	17,021,129	16,418,565	21,868,342	20,091,122	18,195,703
The percent of all data mapping to genome	71.31%	69.08%	69.59%	67.85%	87.23%	87.88%	83.84%	71.58%	86.47%	86.32%	85.95%	70.78%
Unique mapping data	25,997,362	26,362,597	25,777,940	23,914,692	15,536,386	17,602,364	17,884,295	12,917,635	14,049,829	18,288,123	17,027,689	13,331,423
The percent of unique mapped data	54.32%	51.02%	51.30%	50.26%	74.61%	75.54%	71.91%	54.32%	73.99%	72.19%	72.84%	51.86%

* The abbreviation of blossomy petal is BP; ** the abbreviation of wilting petal is WP.

## Supplementary Materials

Supplementary Materials can be found at https://www.mdpi.com/1422-0067/20/23/5949/s1. **Figure S1.** Expression profiles of 30 flower-specific candidate genes from five different tissues by qRT-PCR. Tissues from five plants were collected together, three technical repeats were performed and three biological repeats were conducted. The *B. napus β*-*actin* gene (*AF111812*) was used as a reference standard. R (root), S (stem), L (leaf), F (flower bud), SQ (silique). The asterisk indicates that the expression value in the flower bud was at least three times higher than that in other tissues. **Figure S2.** Histochemical staining of *FSP061::GUS* transgenic *A. thaliana* plants. There is no GUS staining in the two-leaf stage (**a**), six-leaf stage (**b**), rosette leaf (**c**), silique (**i**), or seed (**j**). GUS activity driven by *FSP061* promoter was observed in flower bud (**d**), flowers one day before blooming (**e**), flowers at the blooming day (**f**), petals one day after blooming (**g**), and petals two days after blooming (**h**). Moreover, there is the highest GUS activity in the stamen/stigma (**f**) and less activity in the petals (**f**, **g**, and **h**). Bar = 1 mm. **Table S1** Tissue-specific gene data of 12 different tissues from *Brassica napus*. **Table S2** Transcriptome data of 249 flower-specific candidate genes from 12 different tissues. **Table S3** Primers used in this study.

## Abbreviations

SSR	Sclerotinia stem rot
BP	Blossomy petal
WP	Wilting petal
FPKM	Fragments per kilobase of transcript per million mapped reads
GO	Gene Ontology
Qrt-PCR	Quantitative real-time PCR
4-MU	4-methylumbelliferone
