# Supplementary material for: Identification of Flower-Specific Promoters through Comparative Transcriptome Analysis in Brassica napus
Source: Int J Mol Sci. 2019 Nov 26;20(23):5949. doi: 10.3390/ijms20235949 (PMC6928827; doi:10.3390/ijms20235949)
Supplement: Supplementary file 1 [file ijms-20-05949-s001.zip › ijms-644350-SI-to conversion/Table S1.docx]

| **Table S1.** Tissue-specific gene data of 12 different tissues from *Brassica napus* | | | | | | | | | | | |
| --- | --- | --- | --- | --- | --- | --- | --- | --- | --- | --- | --- |
|  | **Root** | **Leaf** | **Stem** | **Bud** | **Silique** | **Pistil** | **Stamen** | **Petal** | **Sepal** | **Ovule** | **Pericap** |
| Specific gene | 828 | 237 | 38 | 1074 | 166 | 80 | 123 | 397 | 143 | 852 | 101 |
| Known function | 720 | 199 | 25 | 909 | 130 | 62 | 95 | 311 | 111 | 664 | 76 |
| Unknown function | 108 | 38 | 13 | 165 | 36 | 18 | 28 | 86 | 32 | 188 | 25 |
