# Supplementary figures and images for: Identification of Flower-Specific Promoters through Comparative Transcriptome Analysis in Brassica napus
Source: Int J Mol Sci. 2019 Nov 26;20(23):5949. doi: 10.3390/ijms20235949 (PMC6928827; doi:10.3390/ijms20235949)

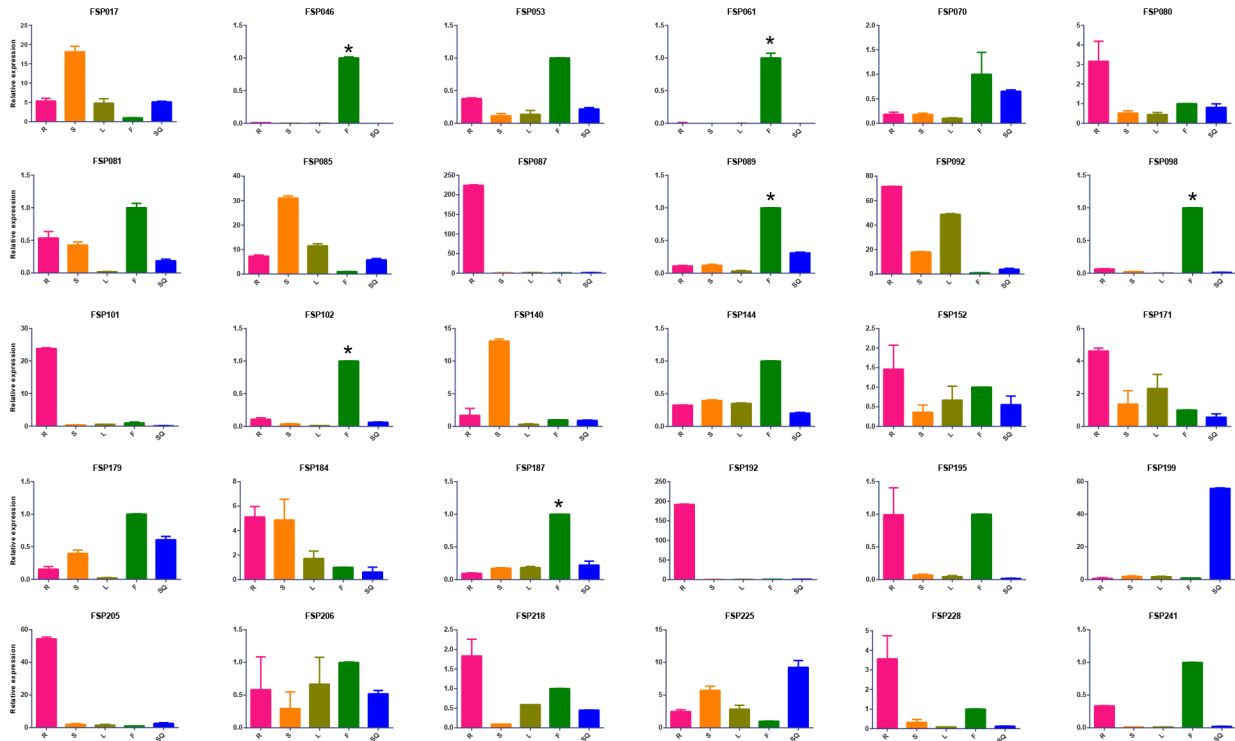

Supplement: Supplementary file 1 [file ijms-20-05949-s001.zip › ijms-644350-SI-to conversion/Figure S1 30 genes expression-1.pdf]

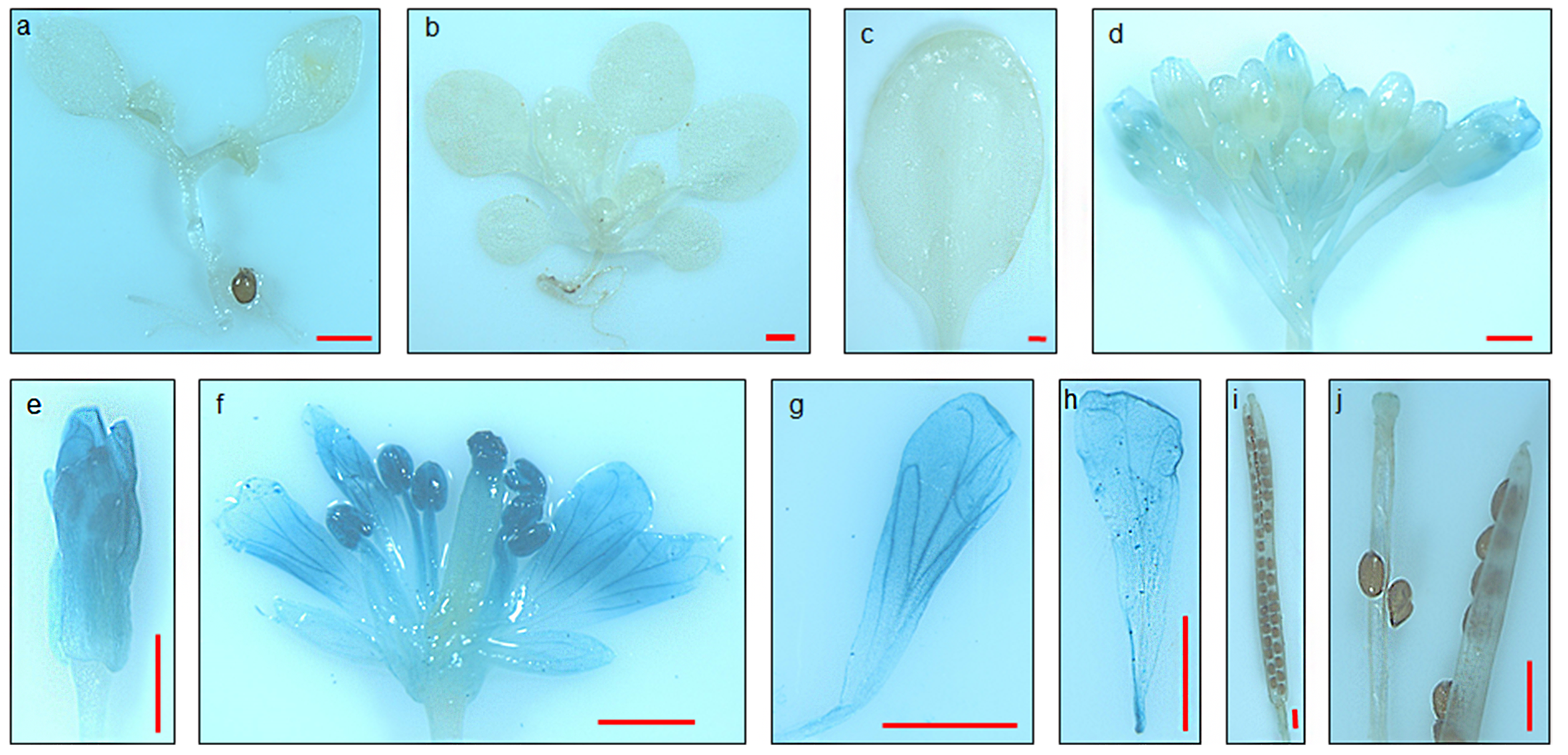

Supplement: Supplementary file 1 [file ijms-20-05949-s001.zip › ijms-644350-SI-to conversion/FigureS2 FSP061p.tif]
